# The Different Colors of mAbs in Solution

**DOI:** 10.3390/antib10020021

**Published:** 2021-05-24

**Authors:** Alexandre Ambrogelly

**Affiliations:** Pharmaceutical and Biologics Operations, Gilead Sciences, 4010 Ocean Ranch Blvd, Oceanside, CA 92056, USA; alexandre.ambrogelly@gilead.com

**Keywords:** monoclonal antibody, color testing, United States Pharmacopeia USP<631>, USP<1061>, European Pharmacopoeia EP2.2.2, tristimulus colorimetry, tryptophan oxidation, kynurenine, N-formylkynurenine, Advanced Glycation End products (AGE)

## Abstract

The color of a therapeutic monoclonal antibody solution is a critical quality attribute. Consistency of color is typically assessed at time of release and during stability studies against preset criteria for late stage clinical and commercial products. A therapeutic protein solution’s color may be determined by visual inspection or by more quantitative methods as per the different geographical area compendia. The nature and intensity of the color of a therapeutic protein solution is typically determined relative to calibrated standards. This review covers the analytical methodologies used for determining the color of a protein solution and presents an overview of protein variants and impurities known to contribute to colored recombinant therapeutic protein solutions.

## 1. Introduction

Proteins in solution have inherent spectrophotometric properties. Protein Absorbance maxima in the UV spectrum at 214 nm and 280 nm, caused by the protein amide backbone and the presence of tryptophan, tyrosine, and cystine residues, are typically used for protein concentration determination and detection after chromatographic separation [1]. Proteins also have an inherent intrinsic fluorescence fingerprint, predominantly derived from tryptophan residues with a typical maximum excitation at ~280 nm and maximum emission at ~350 nm. The spectroscopic properties of tryptophan are complex and highly sensitive to the local structural environment [2]. Modification of the excitation or emission wavelength maximum and intensity are indicative of perturbations in the secondary and tertiary structures of a protein. A variety of spectroscopic analytical methods take advantage of the interaction of light with proteins to derive structural information. These include Circular Dichroism, Fourier-transform infrared spectroscopy, and light scattering to name a few [3].

## 2. Spectrophotometric Properties of Proteins in Solution in the Visible Range

The most noticeable evidence of the spectrophotometric properties of protein solutions manifests itself in the visible spectrum. The human eye is capable to detect light ranging from about 400 to 700 nm which corresponds to the colors going from blue to red, with a peak sensitivity in the yellow range (560 to 590 nm). The natural yellowish tint of proteins in solution is therefore particularly perceptible during visual observation test.

**Figure 1:**
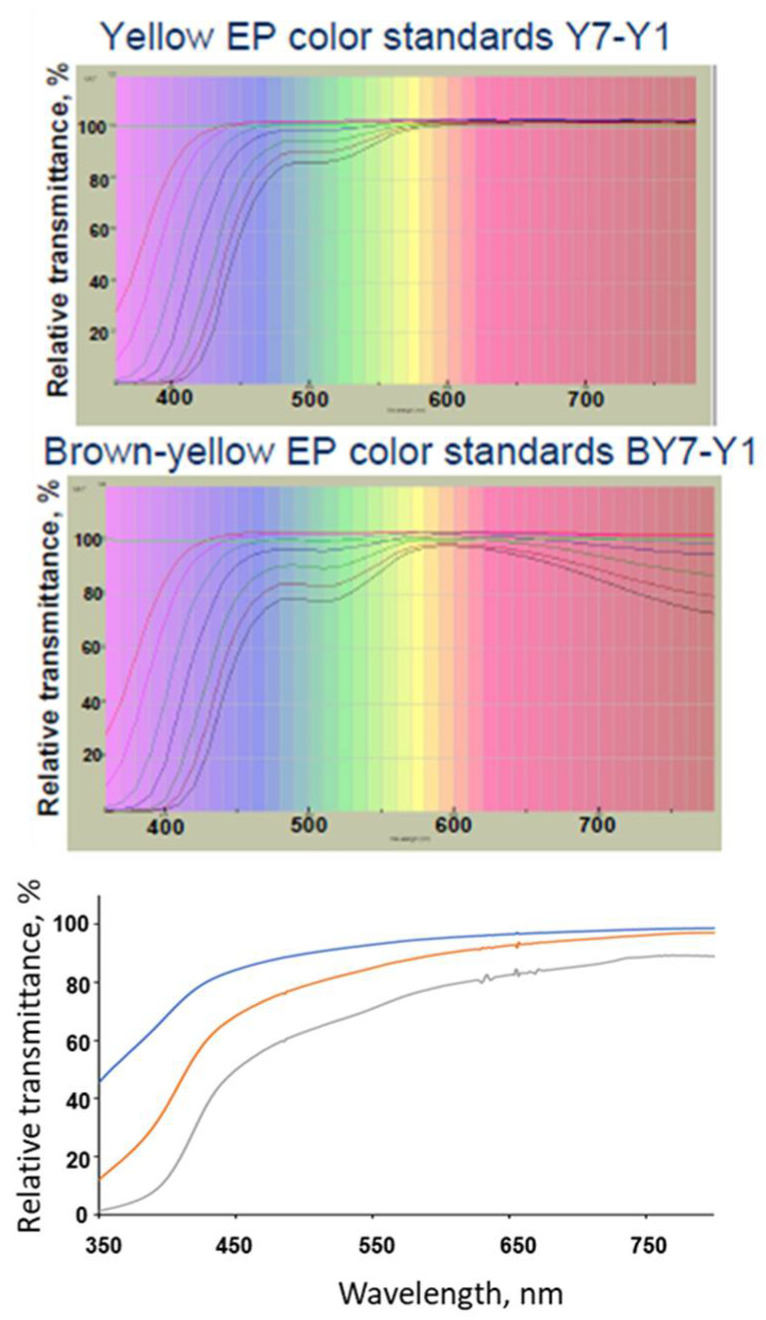
(**Top** panels) Transmittance spectra of Yellow (Y7-Y1) and Brown-Yellow (BY7-BY1) EP standards. (**Bottom** panel) transmittance of non-stressed colorless mAb solutions at 13.5 mg/mL (blue trace), 50 mg/mL (red trace), and 135 mg/mL (gray trace). Adapted from reference [4], top panels reproduced with permission from HunterLab.

The observed yellow color of proteins in solution corresponds to the fraction of white light reflected after the violet/blue components have been absorbed. The protein aromatic residues are chiefly responsible for the absorbance at the lower range of the visible light spectrum as it extends into the violet wavelengths (Figure 1). The intensity of the yellow coloration varies from protein to protein, increases with the number of the tryptophan and tyrosine residues in the sequence, and with protein concentration in solution (Figure 1).

Although a slight yellow tint is the most commonly reported coloration in preparations of purified proteins, other distinctive colors have been documented in the literature. These colorations are always due to the presence of naturally occurring intrinsic or extrinsic chromophores. Perhaps the most widely known type of intrinsic chromophore is the one formed by the posttranslational modification of three amino acids at the core of the green fluorescent protein [2]. In contrast, hemoglobin and photosystems I and II are well documented examples of proteins deriving their color in the visible spectrum from a porphyrin based extrinsic chromophore [5].

Changes of color and color intensity have also been observed for recombinant therapeutic proteins. These changes in the spectrophotometric properties in the visible spectrum often coincide with the presence of protein variants or impurities. We review here the current analytical practice for monitoring and recording the color of protein solutions and present literature reports documenting the identification of the intrinsic and extrinsic chromophores at the origin of color in mAbs and therapeutic proteins (Table 1).

## 3. Compendial and Characterization Methods for Determination of the Color of Protein Solutions

Methodologies for measuring the color of protein solutions are governed by guidance documents published in the European and US pharmacopeia; the Japanese pharmacopeia including elements of both [23,24,25]. European and US pharmacopeia both recommend comparing the color of a protein test article against a series of standards. The US pharmacopeia standards are obtained by diluting cobalt, ferric, and cupric stock solutions with water in set proportions. The resulting 15 individual solutions cover a large part of the visible spectrum, including green, red, yellow and brown yellow series. The EP2.2.2 document describes a similar visual color scale of standards derived from three primary solutions. These solutions when combined and diluted with hydrochloric acid make 37 liquid color standards in five color series: 9 brown, and 7 for brown yellow, yellow, green yellow, and red (B, BY, Y, GY, R) [26]. For each series, standards are given a number, appended to the color series letter, to reflect their relative intensity. The lower the number, the lower the dilution factor, and thus the stronger the intensity (e.g., BY1 is more intense than BY2). BY and Y, commonly used for mAb solutions specifications as the transmittance signature, resembles these two-color series standards used in the European compendial method (Figure 1).

Both European and US compendia allow for the determination of a matching color by analyst visual observation [23,24]. In contrast to the USP compendia, which does not describe the volume and vessel to be used for the color matching test, the European compendia is rather prescriptive. Colorless, transparent, flat base neutral glass sample containers with internal diameter of 15 mm to 25 mm, and a fixed depth of the liquid layer in the tube of 40 mm are to be used. Alternatively, when sample volume is limited, 2 mL glass containers with an external diameter of 12 mm are acceptable. To minimize the impact of the variability induced by the preparation of each individual reference solution, premade sealed ampules with the adequate specifications of the EP standards are commercially available. When prepared manually, reference solutions must be made immediately prior to conducting the test; storing the reference solutions protected from light in sealed ampules with identical dimensions to that used for the test article is acceptable.

To reduce the subjectivity of the exercise prescriptive conditions under which the test may be conducted are described. As the perception of color and color matches is dependent on conditions of viewing, the spectral energy of the illumination, and the visual acuity and sensitivity of the observer, care should be taken to use uniform illumination, minimizing shadows and against a white background. The EP compendia specifies that the viewing must be done horizontally. Both European and US compendia consider diffused natural or artificial daylight as the light source of choice for the comparison between the test article and the color standard. Although neither compendia specify the intensity or the nature of the artificial light to be used, artificial daylight fluorescent lamp combining light in the visible and UV spectrum similar to the D65 emission standards defined by the International Commission on Illumination (ISO 18909:2006) are typically used in manual inspection hoods used for pharmaceuticals color matching. The same D65 light source is recommended in the ICHQ1B guidance document for photo-stability testing of drug substance and drug products [27].

None of the compendia specify requirements on the visual aptitude of the observer. This aspect is left at the discretion of each sponsor and should therefore be addressed and documented as part of analyst GMP training.

Despite the lack of harmonization of the visual observation test, the FDA will accept data derived from compendia from other geographical regions provided the test is equivalent or superior to the corresponding USP test [28]. Given the high degree of convergence between the EP and USP compendial visual observation tests, submission to the FDA may therefore have color determination results generated using the EP compendia. European health agencies recommend using EP2.2.2 but may accept results generated by alternate methods as long as they lead to the same pass/fail result. It is the responsibility of the sponsor to demonstrate the suitability of the alternate methods and approval of the competent authority is necessary in many cases.

With all these assay controls in place, color determination by visual observation is generally suitable to ensure its intended purpose as part of the therapeutic protein control strategy, provided adequate phase appropriate acceptance criteria are set. For low color intensity solutions in particular, acceptance criteria need to reflect the fact that human visual acuity is not sensitive enough for this region of the color space. Indeed, for color intensities higher than 6, color determination by visual observation is highly variable. Pack et al. put the European standards to the color matching test by visual observation by ten trained analysts using the two sample volumes allowed by EP2.2.2 [29]. The 2 mL glass inspection vessel yielded error rate of 80% and above for almost all color series standards with intensity of 6 and 7. Error rates were significantly lower (~50%) for the same diluted solutions when the larger test volume was used. While no literature exists on the USP standards, it is reasonable to assume that under the same experimental conditions a similar outcome would be reached. Color series intensities of 7 and above are virtually indiscernible by visual observation and correspond to essentially colorless protein solutions. These low intensity reference colors should therefore be considered equivalent when used in visual observation [26].

To address the subjectivity of the visual observation methodology, increase the color range detected, as well as being able to detect accurately subtle changes, USP<1061> details the use of quantitative colorimetric methodologies [30]. Efforts towards harmonization of an instrumental method for color determination between the USP, EP and JP pharmacopeia led by the Pharmacopeial Discussion Group have been successful [31]. EP2.2.2 chapter has been revised to include the instrumental method; the revised chapter was published in July 2020 and has become effective on 1st Jan 2021 [32].

**Figure 2:**
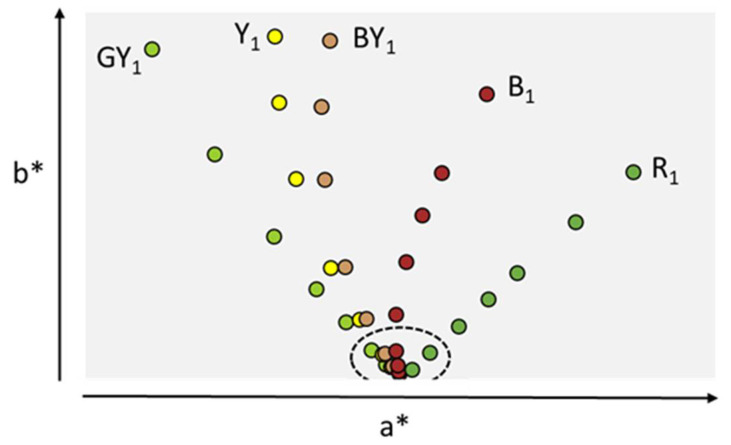
Instrumental determination of a* and b* parameters for EP standards plotted in Cartesian 2D spectral graph. GY stands for Green-Yellow, Y for Yellow, BY for Brown-Yellow, B for Brown, and R for Red. Highest chroma (Intensity 1) for each hue (color series) is shown. Dotted line circle indicates color intensities equal to 6 and above in each color series, not distinguishable by visual observation. Data (a*, b*) are from reference [26].

The instrumental method described in USP<1061> and revised EP2.2.2 is based on industry standard tristimulus colorimetry described in the Commission Internationale de l’Eclairage (CIE) recommendations. The instrumental approach allows the removing of the three main sources of observation subjectivity: (i) observer perception, (ii) illumination source variability and (iii) variable relative position of the sample relative to the source. The use of an instrument allows the tight control of the nature of the light source as well as the position of the sample in the instrument. The subjective color perception of the observer is replaced by sensors producing a weighted aggregated signal modeled on the physiological response of the three cones of an average human eye across the visible spectrum. The reflected light produced by the protein sample is treated and transformed using an observer function algorithm embedded in the instruments’ software. The mathematical treatment of the light signal produces three numerical values L*, a* and b* representing the objective sample color Cartesian coordinates in a 3D color map [26,33]. The L* value indicates the level of light or dark, the a* value redness or greenness, and the b* value yellowness or blueness. Change in hue is most noticeable by the human eye followed by change in intensity of a color, changes in lightness (L) being the least noticeable [33]. For ease of representation, only a* and b* parameters are therefore typically plotted in a 2D spectral graph (Figure 2).

Color consistency, relative to an initial stability sample or an internal standard can be represented as a single composite numerical value derived from the aggregation of the change of the individual L*, a* and b* values [26,33]. For late stage development, acceptance criteria on the maximum allowable color variation composite can be set for the purpose of specification or comparability [33].

USP<1061> allows the use of CIE compatible colorimeters and spectrophotometers, the two types of equipment differing by the degree of sophistication of the detection optics. While colorimeters use three filters (red, yellow, blue), spectrophotometers record reflectance or transmittance from 380 nm to 770 nm at intervals of 10 nm or less. Suitable equipment manufacturers suggested by USP<1061> include BYK-Gardner and Hunter Lab [30].

Validation of the tristimulus colorimetry approach in accordance to ICHQ2 (R1) guidance showed instrument, assay repeatability and intermediate precision of the method to be well below what is considered a visually perceptible color difference [34]. Under the experimental conditions, the spectral method was determined to yield results at least equivalents to those generated by visual assessment [34].

## 4. Color of a Protein Solution Is a Critical Quality Attribute Potentially Caused by Variants or Impurities

Color is a critical quality attribute of therapeutic protein drug substance and drug product as it may relate to the presence of a variant or an impurity. It is typically measured at the time of release and during stability as part of clarity, opalescence and coloration against phase appropriate preset acceptance criteria [35,36]. Failure to meet the preset acceptance criteria would likely result in batch rejection for release, reduced product shelf life claims, and at minima warrant a thorough investigation and justification for comparability.

Although often difficult to investigate, the source of coloration of purified therapeutic protein solutions has in some instances been identified. Two common causes typically account for stronger coloration or an altogether different hue than the expected light yellowish tint. The first one is the modification of one or more of the protein amino acids resulting in a posttranslational modification with photo-spectrophotometric properties. The second consists usually in the formation of an adduct between the recombinant protein and an extrinsic chromophore most often originating from the cell culture broth.

### 4.1. Postranslational Modifications Resulting in an Intrinsic Chromophore

One of the most documented sources of coloration of purified protein solution is the oxidation of tryptophan residues [37]. Tryptophan side chain tends to be buried as they ensure the cohesion of the hydrophobic core of a protein and thus are fairly protected from any environmental aggression. This is particularly true in IgG antibodies as the beta sheet structure and inter-chains interactions are maintained by an important hydrophobic interaction component [38]. However, when the tryptophan residues are part of the CDRs and thus exposed to the solvent or under significant oxidative stress, degradation may ensue. Oxidative stress in cell culture and exposure to visible and UV light during protein handling, manufacturing and storage promote the formation of reactive oxygen species (ROS) and ultimately the radical-mediated oxidation of tryptophan residues [6,7,8,37]. An increase in tryptophan oxidation level from 2% to 6% induced by cell culture components was sufficient to change the color of a mAb solution from pale yellow to light brown [8]. Oxidation of exposed tryptophan and change of color were also documented in formulated therapeutic proteins stability studies at elevated temperature [6]. In some instances, the formation of peroxides through the autoxidation of polysorbate surfactants was shown to accelerate the oxidation process [6,21]. Tryptophan oxidation kinetics depend on a number of factors such as pH, presence of metal ion, nature of the ROS species as well as the nature of neighboring amino acid side chains [6,9].

**Figure 3:**
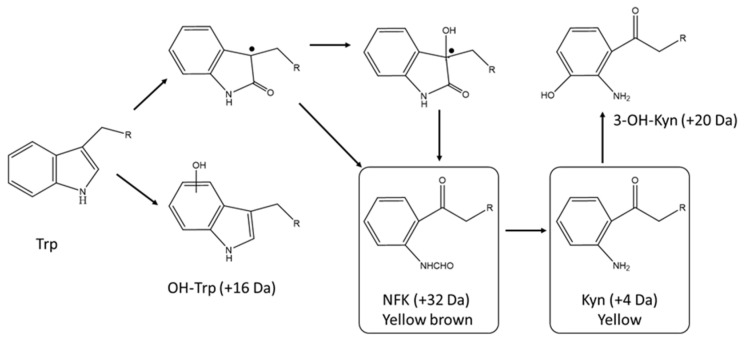
Tryptophan degradation pathways. Trp is for tryptophan, Kyn is for kynurenine, NFY is N-formylkynurenine, OH-trp and 3-OH-Kyn are for hydroxyl tryptophan and hydroxyl kynurenine, respectively. Changes in mass for each tryptophan degradation products are shown. Adapted from reference [6].

The oxidative degradation of the protein tryptophan pathway is fairly well understood as it resembles the initial steps of the well characterized catabolism chemistry of the free amino acid [6,39]. Tryptophan degradation consists in a series of oxidation steps leading to indole ring opening and deformylation. The main degradation products resulting in browning and yellowing of a protein solution are hydroxytryptophan, N-formylkynurenine (NFK), kynurenine (Kyn), and 3-hydroxykynurenine (Figure 3).

**Figure 4:**
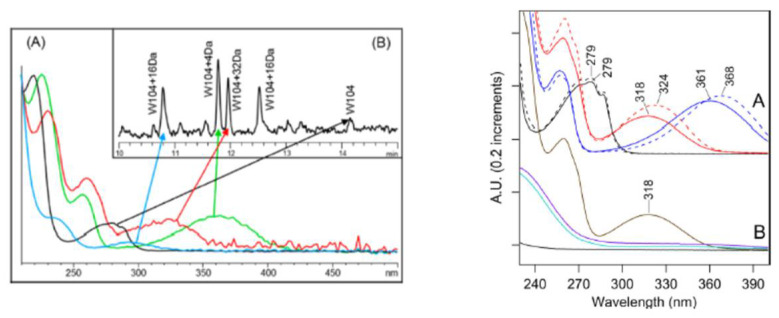
(Left panel, **A**) UV absorption spectra of the peptide containing non oxidized Trp (Black) and oxidized Trp. Kyn (+4 Da, green), NFK (+32 Da, red) and hydroxyl tryptophan (+16 Da, blue) from forced degraded mAb sample (Left panel, **B**) peptide elution profile covering these peptides (A310 nm). (Right panel **A**) Absorption spectra of 40 µM tryptophan in water (black, solid line) and in 50% acetonitrile, 0.1% TFA (black, dashed line). Spectra of 40 µM kynurenine in water (blue, solid line) and in 50%acetonitrile, 0.1% TFA (blue, dashed line). Spectra of 40 µM NFK in water (red, solid line) and in 50% acetonitrile, 0.1% TFA (red, dashed line), Values shown on each curve indicate wavelength at local absorbance maximum. (Right panel **B**) Spectra of a mixture of 40 µM NFK and 160 µM BSA (brown) and when treated with 400 µM NaBH_4_ in water (cyan). Spectrum of 160 µM BSA when treated with 400 µM NaBH_4_ in water (black) and spectrum of 40 µM NFK when treated with 400 µM NaBH_4_ (violet) in water. Reproduced with permission from reference [6,41].

Analytical methodologies based on combinations of chromatographic separation, mass spectrometry and spectrophotometric approaches have been developed to monitor the formation of tryptophan degradation products in proteins [6]. Controlled enzymatic digestion with specific peptidases followed by reversed phase chromatography reveal the presence of degraded tryptophan in peptides owing to their earlier retention time and decreased absorbance at 280 nm relative to the corresponding intact peptides (Figure 4) [6]. Each tryptophan degradation product was shown to have its specific spectrophotometric signature: the formation of Kyn or NFK results in an increase of peptide absorbance at 365 nm and 320 nm, respectively (Table 2) [6,40,41]. The presence of Kyn in peptides results in a more intense and darker yellow color than those containing NFK as a result of its absorbance at higher wavelengths [6]. Oxidation of tryptophan to hydroxytryptophan results in the lowest shift towards higher wavelength and therefore is the least likely to contribute to a protein color change [6]. The absorbance spectrum of peptides containing Kyn and NFK residues resemble that of the free Kyn and NFK amino acids, known to have a yellow and yellow-brown colorations in solution, respectively (Figure 4) [6,40,41].

Degradation of tryptophan residues to Kyn not only modifies the absorbance properties of proteins but also their fluorescence [16,41]. Formation of Kyn results in a shift of the maximum absorption wavelength from 297 nm to 330 nm and 380 nm, and maximum emission intensity wavelength from 348 nm to 480 nm (Table 2) [41]. However, detection of the tryptophan degradation products by florescence is difficult because of the low quantum yield of Kyn and the heterogeneity of the structures formed [39].

Kyn was shown to be sensitive to reduction by sodium borohydride [8,41]. Demonstration of the presence of a Kyn chromophore can be made by comparing the spectrophotometric properties of a molecule of interest before and after reduction (Figure 4) [8,41].

Sequential degradation of tryptophan also induces a relative molecular weight change detectable by LC/MS when present in large enough amounts. Increases of 16 Da, 32 Da, 4 Da, and 20 Da relative to the unmodified peptide coincide with the formation of hydroxytryptophan, Kyn, NFK, and 3-hydroxykynurenine degradation products (Figure 3).

The study of tryptophan sensitivity to oxidation and change in color in therapeutic proteins is performed in a number of ways: forced oxidation with peroxides such as α,α′-Azodiisobutyramidine dihydrochloride (AAPH) at various pHs and in the presence or absence of metal ions favoring the formation and propagation of radicals, exposure to visible and UV light as per ICH Q1B guidance document as well as stability storage at elevated temperatures [27,47].

Tryptophan degradation products Kyn and NFK contribute to yellow, yellow brown color change due to absorbance at wavelengths higher than 280 nm. Oxidation of other amino acids also sensitive to oxidation such as tyrosine, histidine and methionine do not correlate with change of color of the protein in solution [6].

### 4.2. Formation of Covalent Adducts Resulting in Extrinsic Chromophores

Formation of covalent adducts between a protein and a chromophore or chromophore precursor is also a common source of coloration of proteins.

#### 4.2.1. Formation of Advanced Glycation End Products Extrinsic Chromophores

The best-known type is perhaps that of heterogeneous degradation products collectively known as Advanced Glycation End products (AGE) [37]. This phenomenon was first invoked to explain the yellow/yellow brown coloration of aging human lens crystallins, particularly in diabetic subjects [48,49].

**Figure 5:**
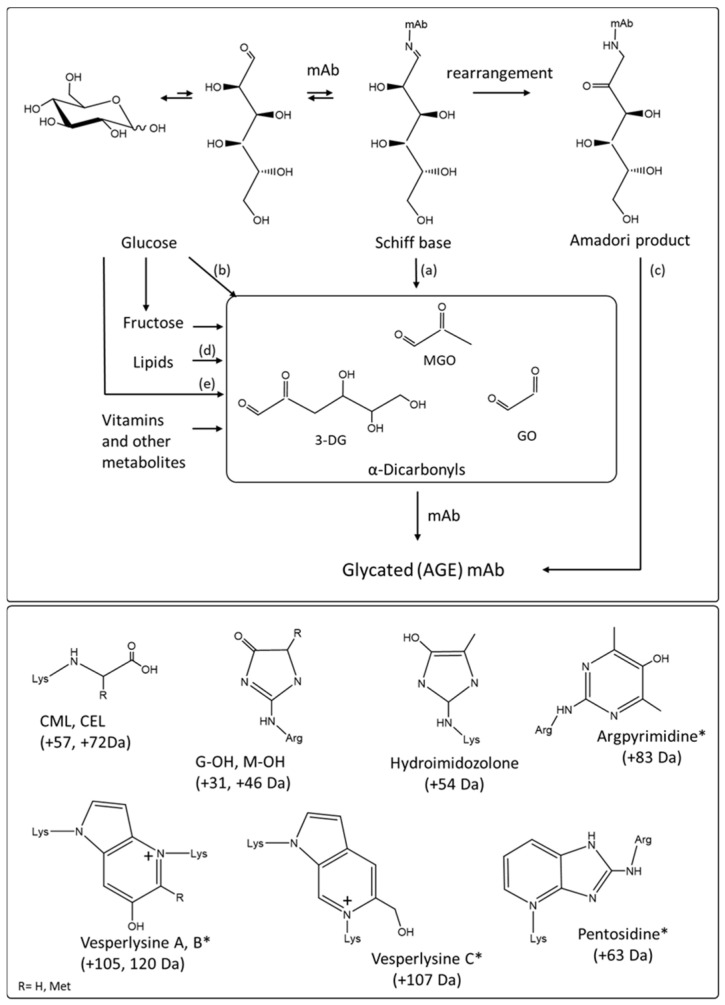
(top panel) AGE products formation through rearrangements, oxidative glycosylation and non-oxidative degradation of the shiff base between a reducing sugar and the reactive side chain of a protein amino acid (lysine, arginine), and through formation of dicarbonyl products through oxidative and non-oxidative degradation of carbohydrates. (**a**) Namiki pathway (**b**) oxidative oxidation, (**c**) oxidative and non-oxidative pathways (**d**) peroxidation (**e**) non oxidative pathway. 3-DG stands for 3-deoxyglucasone, GO stands for glyoxal, MGO stands for methylglyoxal. Adapted from Reference [37]. (lower panel) Chemical structure of representative fluorescent (*) and non-fluorescent AGE products derived from lysine and arginine: Nε-carboxymethyl-lysine (CML), Nε-carboxyethyl-lysine (CEL), Glyoxal hydroxyimidazolone (G-OH), Methyl glyoxal hydroxyimidazolone (M-OH), Hydroimidozolone, argpyrimidine, vesperlysines A, B and C, pentosidine. Changes in mass for each AGE degradation product are shown. Spectrophotometric properties of these are shown in Table 2.

The structures of AGE products and their formation pathways are complex and diverse, but all originate from the reaction of a reducing sugar, most often glucose or ribose, or dicarbonyl byproducts of carbohydrate oxidative degradation with a protein amino terminus or with lysine or arginine side chains (Figure 5) [16,17,50,51].

Whether the degradation of the carbohydrate chain occurs once attached or in solution prior to reacting with the protein, the resulting species are highly unstable and will undergo further reactions. These reactions include dehydrogenation, dehydration, cyclization, condensation, isomerization, oxidation, and fragmentation steps leading to simple structures such as carboxyl methyl lysine (CML) to more complex pyroline and pyrimidine ring structures, or to pentosidine and other structures obtained via crosslinking of lysine or/and arginine amino acid side chains (Figure 5) [17,43,52,53].

Although the formation an initial shiff base adduct between a reducing sugar and an amino acid side chain does not change the spectrophotometric properties of the protein, the different degradations and rearrangements of the carbohydrate chain coincide with a progressive shift towards absorbance at higher wavelengths, the AGE products absorbance extending in the blue region of the visible spectrum above 400 nm [16,42,44]. The change of color is accompanied by changes in fluorescence properties [48]. AGE products were found to produce a maximum intensity emission peak at about 430 nm after excitation at 360 nm in yellow/brown lens proteins [31]. Fluorescence using excitation and emission light at 360/430 nm is a generalizable marker for the sensitive detection of AGE posttranslational modifications of proteins [42,45,48].

Fluorescence or immunoassay based approaches are the simplest analytical methods for the detection of AGE products in proteins [51]. In spite of their limitations, these techniques remain routinely used. One such limitation is the potential lack of specificity as other posttranslational modifications such as tryptophan degradation can indeed contribute and interfere with the AGE product fluorescence emission measured at 430 nm. In addition, not all AGE products are fluorescent (Figure 5). Although the number of anti-AGE monoclonal and polyclonal antibodies commercially available regularly increases, the specificity of these antibodies is usually restricted to a handful of chemical structures. The most commonly used antibodies as marker for the formation of AGE products are directed against CML or pentosidine for instance [51]. As neither fluorescence and immunoassay-based methods allow identifying the specific structures responsible for coloration nor their location in the protein they are therefore used primarily as screening tools.

Despite the complexity, variety and low abundance of the potential colored protein-AGE product structures, mass spectrometry is the method of choice for their identification and quantification [16,43,54]. Separation methods based on charge offers the opportunity to isolate protein variants enriched in AGE products owing to their more acidic isoelectric point caused by the modification of lysine and arginine residues, and facilitate identification [16,17,18,19]. In a number of reported cases, mass spectrometry derived results have been shown to correlate well with protein fluorescence emission at 430 nm and immunoassay tests [16,55].

Descriptions of AGE products in mAbs are as rare as the studies on the impact on glycation are numerous. To date, CML has been described in a single color changed therapeutic monoclonal antibody [16]. In this report CML content was measured at up to 1% and shown to correlate with AGE structures related fluorescence signature determined by normalized intrinsic fluorescence [16]. Carboxyethyllysine, imidazolone modifications of arginine, and vesperlysines were also detected in the most acidic fractions of two mAbs albeit to levels well below 1% [16,18]. In a different study, the modification of adalimumab with a byproduct of ascorbic acid known to lead to formation of the fluorescent structures such as versperlysine and pentosidine was also described [19,20]. While AGE products description in mAbs remain scarce, glycation sites leading to the formation of potential colored AGE products have been widely studied [37]. Exposed and reactive lysine residues in framework of the variable domain and in conserved domains of mAbs have been mapped via forced glycation studies [17,54].

The cell culture conditions with a neutral pH, oxidative environment, and glucose and ribose based vitamins necessary to sustain cell growth are particularly conducive to glycation and the formation of AGE products [56]. Glycation has also been documented during stability studies at elevated temperatures because of the degradation and rearrangement of the sucrose bulking agent typically included in formulations as well as upon dilution into dextrose during IV infusion [17,22].

AGE derived chromophores responsible for the yellow/brown color have not been frequently reported in mAbs [17]. This perceived low incidence might in part be the result of abundance typically lower than the detection limit of most analytical methods and the inherent difficulty to identify, detect and quantify such diverse chromophores.

#### 4.2.2. Formation of Extrinsic Chromophores Originating from Cell Culture Components

The cell culture broth and feeds contain a wide variety of components in non-limiting quantities necessary to sustain the rapid growth and high productivity of the cells. Vitamins are amongst these critical key components. Vitamins are often colored and by nature reactive. Molecules in this class of compound are therefore prime candidates for forming colored adducts with the recombinant protein being expressed.

**Figure 6:**
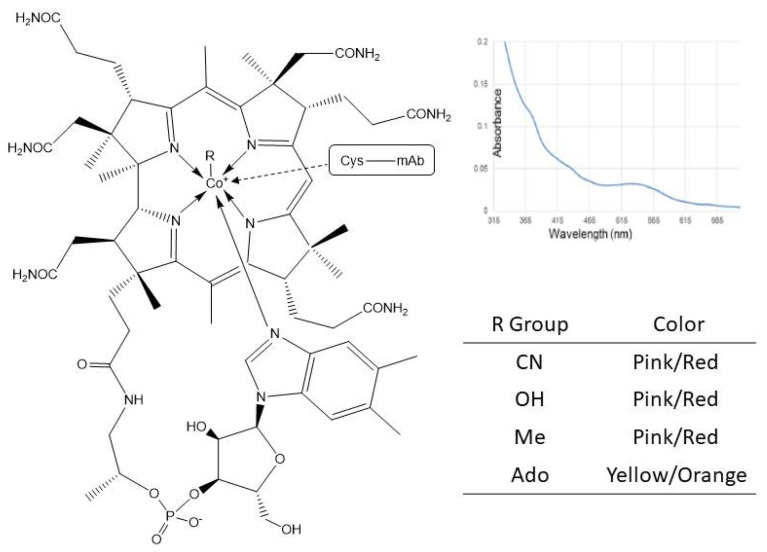
Vitamin B12 structural variants and associated color. (right panel) Absorption spectrum of a purified pink colored mAb preparation. Local maximum at about 365 nm reflects the presence of vitamin B12 (hydroxocobalamin). Reproduced with permission from reference [12].

Association of vitamin B12, in its hydroxocobalamin form has been reported in a number of instances to contribute to a red/pink coloration of mAbs of different isotypes and Fc-Fusion purified material [8,10,11,12]. Red colored mAbs were found to have absorbance at about 365 nm, which corresponds to one of the absorbance maximum of vitamin B12 (Table 2, Figure 6). In addition, the red coloration correlated with higher cobalt ion concentrations in the protein sample as determined by ICP/MS. A cobalt ion is coordinated at the core of porphyrin structure of the B12 vitamin; therefore, cobalt concentration is a good indirect marker of the association with a recombinant protein (Figure 6).

The mode of association between vitamin B12 varies from protein to protein. Different reports suggest a positive correlation between color and reduced disulfide bridges content but ascribe different roles to the generated free thiols. For some mAbs, free thiols may just be markers of unfolded mAb domains, exposing to the surface hydrophobic/charged residues for non-covalent binding of the vitamin [8,11]. In other mAbs, LC-MS/MS data suggest a more covalent nature to the association as a protein free thiol enters the coordination sphere of the vitamin B12 cobalt ion [12]. In either case, both free thiols and elevated vitamin B12 concentration factors are necessary but neither one is sufficient alone to generate protein coloration.

As for other chromophores, low levels of modification are sufficient to impart coloration to the protein. A molar ratio of 1 to 50 (vitamin B12 to mAb) was enough to give a distinct red/pink coloration to the purified protein solution [12].

Non-covalent association between IgGs and IgAs from normal human serum and the flavin ring of flavin adenine dinucleotide (FAD) and its biochemical precursor vitamin B2 (riboflavin) were also reported [13,14]. The dissociation constants of FAD and riboflavin in the presence of pooled human plasma immunoglobulin fractions were estimated to be in the low nM range, reflecting much higher affinities than to other components of the serum such as albumin [14]. This observation makes a subset of the human immunoglobulins natural binders and carriers of flavin containing cofactors. The flavin ring binding site was identified by structural characterization of two bright yellow colored antibodies isolated from patients with yellow pigmented skin developed during the progression of multiple myeloma [15]. The binding site was shown to be located in the variable portion of the antibody with residues from three heavy chain and one light chain CDR loop making contact with the flavin ring [15].

## 5. Conclusions

Detecting and monitoring color evolution by visual inspection of protein samples is a difficult task. Continuous improvement of instrumentation as well as evolution and harmonization of the regulatory landscape across all regions has opened the door to standardized instrument-based determination of color consistency in biologics products.

## Figures and Tables

**Table 1:** Factor potentially inducing color change in proteins.

Factor	Degradation Observed	Color Observed	Reference
UV light exposure	Tryptophan oxidation	Yellow/Yellow Brown	[6,7]
Copper/Iron in cell culture	Tryptophan oxidation and AGE products ^1^	Yellow/Yellow Brown	[8,9]
Vitamin B12 (hydroxocobalamin) in cell culture	Adduct	Red/Pink	[8,10,11,12]
Vitamin B2 (riboflavin) in cell culture	Adduct	Yellow	[13,14,15]
Glucose/Ribose and dicarbonyls in cell culture	AGE products	Brown	[16,17,18]
Vitamin C in cell culture	AGE products	Brown	[19,20]
Sucrose in formulation	AGE products ^1^	Brown ^2^	[17]
Polysorbate excipient in formulation	Tryptophan oxidation	Yellow/Yellow Brown ^2^	[6,21]
Dextrose during infusion	AGE products ^1^	Brown	[22]

^1^ Degradations expected, not formally identified. ^2^ Color expected based on degradations reported.

**Table 2:** Modification of intrinsic fluorescence properties of oxidized tryptophan amino acid, and AGE product with absorbance spectrum extending in the blue region of the visible spectrum.

	Excitation Maximum Wavelength	Emission Maximum Wavelength	Reference
Tryptophan	280 nm	348 nm	[2,40]
Kynurenine (Kyn)	330 nm and 380 nm	480 nm	[40]
N-formylkynurenine (NFK)	325 nm	434 nm	[16]
Vesperlysine	380 nm	440 nm	[16,42]
AGE products	360 nm	430 nm	[43,44,45]
Pentosidine	335 nm	385 nm	[46]
Vesperlysine A, B	366 nm	442 nm	[46]
	**Absorbance Maximum Wavelength**		
Tryptophan	280 nm		[6,40,41]
Kynurenine (Kyn)	260 nm and 365 nm		[6,40,41]
N-formylkynurenine (NFK)	260 nm and 320 nm		[6,41]
Hydroxy tryprophan	240 nm and 295 nm		[6]
Vesperlysine	302 nm and 363 nm		[16,42]
AGE products	330 nm		[42,43,44]
Vitamin B12	360, 420, 550 nm		[12]

## Abbreviations

UV: Ultra Violet, CD: Circular dichroism, FTIR: Fourier-transform infrared, FDA: Food and Drug Administration, EP: European pharmacopeia, USP: US pharmacopeia, JP: Japanese pharmacopeia, ICH International Council for Harmonization of Technical Requirements for Pharmaceuticals for Human Use, CDR: Complementarity-determining regions, LC/MS: Liquid chromatography/mass spectroscopy.
